# Effect of trabeculectomy on the rate of progression of visual field damage

**DOI:** 10.1038/s41433-022-02312-y

**Published:** 2022-12-07

**Authors:** Susanna Friederike Koenig, Giovanni Montesano, Clarissa Ern Hui Fang, David Paul Crabb, Hari Jayaram, Jonathan Clarke

**Affiliations:** 1 Moorfields Eye Hospital NHS Foundation Trust, 162 City Road, EC1V 2PD, London, UK; 2 Universitaetsaugenklinik Ulm, Prittwitzstrasse 43, D – 89075 Ulm Deutschland, Germany; 3 Optometry and Visual Sciences, City, University of London, London, UK; 4 NIHR Biomedical Research Centre of Ophthalmology, Moorfields Eye Hospital and UCL Institute of Ophthalmology, London, UK

**Keywords:** Outcomes research, Optic nerve diseases

## Abstract

**Objectives:**

This study quantifies the effect of trabeculectomy on the rate of progression (RoP) of visual field (VF) damage utilising pre- and post-operative visual function as the outcome instead of surrogate outcomes of success.

**Methods:**

Clinical and VF data from 199 sequential patients who underwent trabeculectomy between 2015 and 2016 were extracted from the network of sites of Moorfields Eye Hospital NHS Foundation Trust. Of these, we analysed 80 eyes of 74 patients who met our inclusion criteria of at least three reliable VFs before and after surgery (false positive rate <15%). The change in mean RoP (dB/year) was tested using point-wise sensitivity values through a mixed effect model with random effects on both intercepts and slopes. A broken-stick regression of sensitivity over time, with a breakpoint at the day of surgery, modelled the individual change in RoP.

**Results:**

We analysed 10 [9,12] VFs per subject (Median [Interquartile Range]). At surgery, the age was 67 [57, 72] years, mean deviation was −10.84 [−14.7, −5.6] dB and the IOP was 18 [15, 20] mmHg. One year after surgery, the IOP was 10 [8,13] mmHg (*p* = 0.002). Mean RoP before surgery was −0.94 [−1.20, −0.69] dB/year (Mean [95% credible intervals]) and it was slowed down by 0.62 [0.26, 0.97] dB/year (*p* < 0.001) after surgery.

**Conclusions:**

Trabeculectomy leads to a significant reduction in the RoP of VF loss postoperatively.

## Introduction

The primary goal of glaucoma treatment is to preserve vision and vision-related quality of life. Since knowledge of factors contributing to optic nerve damage and concomitant visual field defects are limited, the predominant way to treat glaucomatous optic neuropathy (GON) is to lower the intraocular pressure (IOP) [1–3]. Landmark glaucoma trials have confirmed the value of IOP-lowering in delaying visual field (VF) progression [1–3]. However, progression is rarely completely stopped surgically or medically.

Progression in glaucoma is described as worsening of functional defects. Optic disc imaging (structural) and visual field testing (functional) are complementary [4]. Various methods exist to track progression of VF damage over time. Trend analyses use a series of measurements to determine the rate of progression of such a damage over time. As a result, they provide estimates of the velocity of vision loss, which can help clinicians perform risk assessments of their patients and forecast how these changes will affect patients’ vision and quality of life.

Trabeculectomy has become the standard filtering operation for medically uncontrolled, progressive glaucoma. Its beneficial IOP-lowering effect has been reported in different studies [5, 6]. However, further deterioration of VF defects has been described after surgery either related to glaucoma or co-pathologies. Previous studies have described further VF deterioration after trabeculectomy in 13–83% of cases [6, 7]. On the contrary, reversal of structural change and field loss in newly diagnosed patients could be demonstrated after commencement of treatment [8]. More recent data have indicated that surgically induced IOP-lowering can also lead to improvement of VF [9, 10].

Wright and al. reported short-term improvement of central and peripheral VF sensitivity after surgical IOP reduction in glaucomatous eyes [11]. Short-term improvement of contrast sensitivity, colour vision testing and electroretinography could also be demonstrated after glaucoma surgery [12–14].

Although IOP reduction and resulting slowing of VF damage have been established in glaucoma randomized clinical trials, the real world effectiveness on the rate of progression has been only partially investigated [2].

In this retrospective study, we evaluated rate of progression of visual field loss before and after surgical IOP lowering by means of primary trabeculectomy without consideration of changes to the optic disc. We aim to better inform clinicians and their patients about the likely outcome of trabeculectomy.

## Methods

### Patients

This retrospective study was approved as a clinical audit by the Clinical Audit Assessment Committee Moorfields Eye Hospital NHS Trust (audit number 550). It adhered to the tenets of the Declaration of Helsinki.

Clinical and VF data from 199 patients, randomly selected from the cohort of patients that underwent trabeculectomy between 2015 and 2016 at the network sites of Moorfields Eye Hospital NHS Foundation Trust, were extracted from clinical charts and digital archives. The Trust annually collects these data retrospectively as part of the “core outcomes” assessment of the main surgical interventions of all sub-specialties.

Patients were either referred by primary care physicians for assessment and management of glaucoma or referred specifically for surgery by ophthalmologists at other institutions who continued their long-term care. Inclusion criteria for analyses were a minimum follow-up of at least one year and ≥ three reliable VFs before and after surgery. Reliability was defined by a false positive rate < 15% as this has been proven to be the most relevant reliability index [15]. Indications for trabeculectomy were medically uncontrolled IOP despite maximum tolerated medical treatment and/or progression of VF. Of these 199 screened patients, 72 (36.2%) were excluded because of insufficient pre-operative VF data, 13 (6.5%) because of insufficient post-operative VF data and 16 (8.0%) due to insufficient pre and post-operative VF data. Eighteen patients (9.0%) were then excluded because they did not have at least three reliable VF tests before and after surgery, leaving 80 eyes (40.2%) of 74 patients with eligible VF data.

### Trabeculectomy surgery for glaucoma

Trabeculectomy with application of mitomycin C (MMC) was performed by 39 experienced surgeons according to the standard operating procedures of the department. A fornix-based approach was performed. After creation of a fornix-based conjunctival flap in an upper quadrant, the surface of the sclera was carefully cauterized using a monopolar device. Mitomycin C was then applied to the sclera at the discretion of the surgeon and subsequently rinsed out using a balanced salt solution. Typically, a 4 mm limbus-based rectangular flap was created through dissection of the sclera, followed by a 1 ×1 mm punch descemetectomy and peripheral iridectomy. Then the scleral flap was repositioned and sutured tightly using 10-0 nylon releasable sutures, which could be adjusted or removed after surgery in order to adjust flap closure and maintain a low IOP.

The number of sutures was the surgeon’s own personal decision. Operations were performed under local or general anaesthesia according to the patient’s requirements and/or the surgeon’s recommendations, as well as the patient’s preference. Eyes with technical variations in surgical procedures in accordance with the surgeon’s clinical decision were also included in our analysis.

### Visual field testing

White-on-white perimetry was performed with the 24-2 pattern using a Humphrey Field Analyzer (HFA) (Zeiss Meditec, Dublin, CA). Examinations had to be either full threshold or SITA Standard/Fast to be included. Each patient was followed-up with the same test strategy. No VF was performed within the first three months after surgery. Refraction-matching lens correction was carried out and visual field testing was performed on each eye separately.

### Statistical analysis

**Fig. 1:**
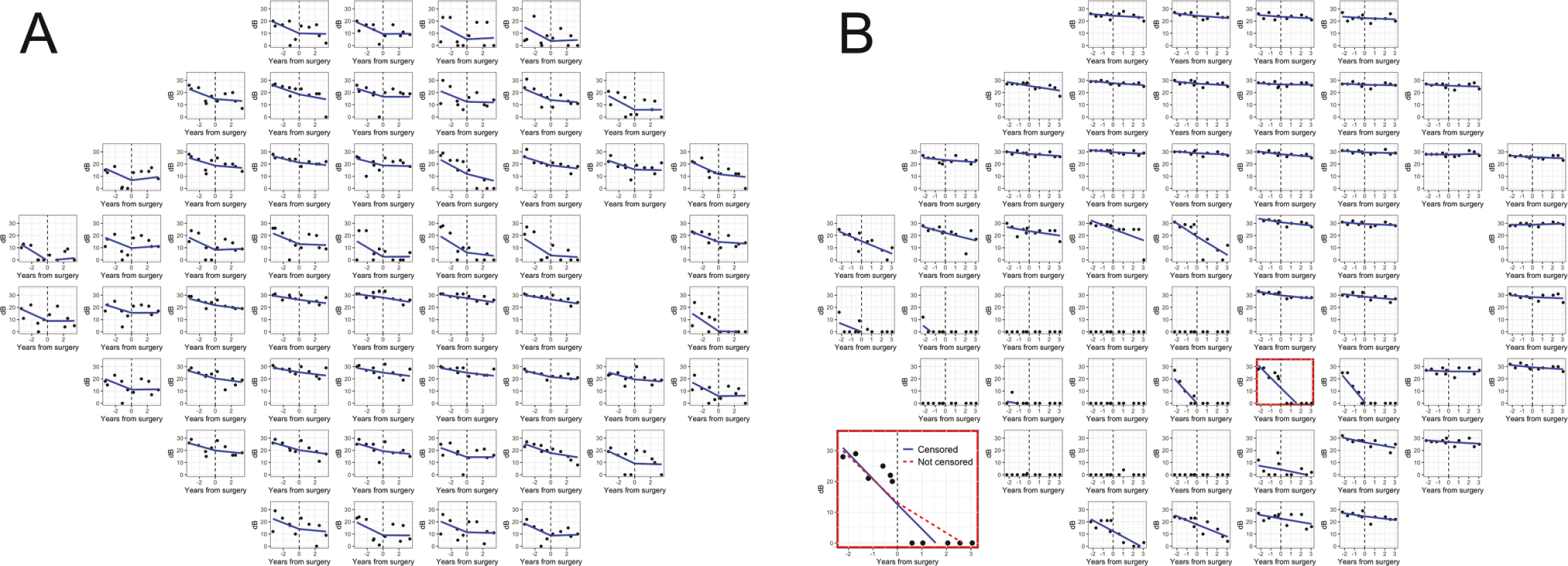
Two examples of the model fit on individual eyes from our data. **A** Eye in which there is evidence of surgery (marked by a vertical dashed line) slowing down the progression of visual field damage. **B** Eye in which surgery does not determine a change in the rate of progression. The small panel (bottom left) highlights a location where not accounting for censored data (red dashed line) would wrongly show a decrease in the rate of progression, inflating the real effectiveness of surgery.

All analyses were conducted in R (R Foundation for Statistical Computing, Vienna) [16]. The change in rate of progression (RoP) was tested using point-wise VF sensitivity values through a mixed effect model with random effects on both intercepts and slopes. We used two nested levels of random effects (patient and location within the VF). The fixed effects modelled progression of VF damage over time at each individual location. The model included a break point at the date of surgery, yielding a two-stage regression (broken-stick model): two different RoPs were estimated for the pre and post-operative period, while forcing the two lines to meet at the day of surgery (Fig. 1). The difference in the population slope before and after the break-point was the outcome of interest, providing an estimate of the change in the RoP after surgery. Because eyes could have advanced VF damage, sensitivity at many locations could be close to the lower measurement floor (0 dB). Trails of 0 dB sensitivity values in a VF series, arising from censored observations, can positively bias the slope estimates, as they appear as locations with a stable sensitivity of 0 dB. Instead, these measurements only provide partial information, i.e. that the actual sensitivity is below the floor, but its value cannot be measured [17]. This is particularly problematic for our analysis because artefactually shallower slopes after the breakpoint might inflate the beneficial effect of surgery (see examples Fig. 1B). To overcome this, we have estimated our mixed effect model through Bayesian computation, so that we could account for censored values [18]. Details of the model are provided as Supplementary Material

Random effects were used to model correlations between observations from the same eye and repeated observations for the same location over time. The use of random effects also allows for the RoP estimates of individual locations to be informed by the general trend of the VF. This is helpful for locations very close to the measurement floor, for which only few observations would be fully informative. Bayesian models do not provide *p*-values, but a similar metric can be derived from the Bayesian P-direction, with essentially the same interpretation as a two-tailed *p*-value [19]. We will refer to this index as p_d_, whereas p will be reserved for the usual *p*-values. The analysis was also repeated with a traditional frequentist approach, without accounting for censoring, using the package *lme4* [https://www.jstatsoft.org/article/view/v067i01] for R and are reported as Supplementary Material. Note that correlations between the two eyes from the same subject were ignored because only 6/74 patients had both eyes included in the analysis. Finally, because Bayesian methods allow inference on random effect estimates, we could calculate a one-sided p_d_ on the change in RoP for each eye, to determine for how many eyes the RoP was significantly slowed down by surgery (i.e., the difference between the pre and post-operative RoP was > 0, with a one-sided p_d_ < 0.05).

Additional clinical parameters were gathered and included from recorded examinations and the given medical history of the patient at listing and one year after surgery: best-corrected visual acuity (BCVA), intraocular pressure (IOP) assessed by means of Goldmann applanation tonometry (GAT), number of topical and systemic IOP-lowering medication and the number of previous glaucoma surgeries. To explore the effect of IOP at one year on the post-operative RoP, we modified the model used for the main analysis to include an interaction term modelling the effect of IOP on the change in RoP (see Supplementary Material).

## Results

Eighty eyes of 74 patients (54.1% female, 67 years [57,72] (median [interquartile range])) with IOP-lowering surgery by means of trabeculectomy with MMC respectively were included into this retrospective study. They all had medically uncontrolled IOP despite maximum treatment and/or VF progression. Trabeculectomy was part of their standard care as recommended by their treating physician. Demographic characteristics as well as data before and after surgery are given in Table 1. Postoperative complications are listed in Table 2.

**Table 1:** Demographics (*n* = 80 eyes).

	Median [interquartile range]
Age (years)	67 [57, 72]
VF per subject (*n*)	10 [9, 12]
Pre-op follow-up period (years)	2.8 [1.8, 4.3]
MD pre surgery (dB)	−10.84 [−14.68, −5.56]
Pre-op IOP (mmHg)	18 [15, 20]
Pre-op topical medication (*n*)	3.0 [1.0, 4.0]
Post-op follow-up period (years)	3.1 [2.7, 3.5]
Pre-op BVCA (logMAR)	0.2 [1.0, −0.1]
Post-op IOP (1 year after surgery; mmHg)	10.0 [8.0, 13.0]
Post-op topical medication (*n*)	0.0 [0.0, 3.0]
Post-op BCVA (logMAR)	0.2 [1.0, −0.2]
Number of patients needing Acetazolamide pre-op (*n*)	3
Number of patients needing Acetazolamide post-op (*n*)	0
Gender (f: m)	40:34
Eyes with crystalline lens (phakic) (*n* (%))	54 (68%)
Eyes with artificial intraocular lens (pseudophakic) (*n* (%))	26 (32%)

*VF* visual field, *MD* mean deviation, *IOP* intraocular pressure, *BCVA* best corrected visual acuity, *dB* decibel.

**Table 2:** Post-op complications (*n* = 80 eyes).

Post-op complications	*n* (%)
Early transient hypotony (resolved without intervention)	12 (15)
Post-op hyphema (resolved spontaneously)	4 (5)
Needling within the first three months post-op (encapsulated bleb)	8 (10)
Bleb leak (resolved medically)	7 (8.75)
Surgical bleb revision within first three months post-op	11 (13.8)
Needling within one year post-op	7 (8.8)
Late hypotony with revision	2 (2.5)
Bleb dysaesthesia	2 (2.5)
Late hypotony (resolved spontaneously)	2 (2.5)
Numerical hypotony	3 (3.75)

Most eyes (*n* = 49, 61.3%) were diagnosed with uncontrolled primary open angle glaucoma (POAG), followed by 27.5% (*n* = 22) normal tension glaucoma (NTG), 5.0% (*n* = 4) having secondary glaucoma (three patients after retinal surgeries, one patient having uveitic glaucoma), two patients (2.5%) having primary angle closure glaucoma (PACG), further two (2.5%) pseudoexfoliation glaucoma (PXFG), and one patient (1.3%) with pigmentary glaucoma. The diagnostic characteristics are also listed in Table 3. Nine patients (11.3%) underwent selective laser trabeculoplasty (SLT) prior to surgery. Median [interquartile range] follow-up period was 2.8 [1.8, 4.3] years before surgery and 3.1 [2.7, 3.5] years after surgery.

**Table 3:** Diagnostic characteristics.

Type of glaucoma	*n* (%)
Primary open angle glaucoma (POAG)	49 (61.3)
Normal tension glaucoma (NTG)	22 (27.5)
Secondary glaucoma	4 (5.0)
Primary angle closure glaucoma (PACG)	2 (2.5)
Pseudoexfolation glaucoma (PXFG)	2 (2.5)
Pigmentary glaucoma	1 (1.3)

Secondary glaucoma: three patients after retinal surgeries, one patient with uveitic glaucoma.

We analysed 10 [9, 12] VFs per subject (Median [interquartile range]). At surgery, the mean deviation (MD) was −10.84 [−14.68, −5.56] dB and the intraocular pressure (IOP) was 18 [15, 20] mmHg on 3.0 [1.0, 4.0] topical agents and two patients needing further systemic acetazolamide. One year after surgery, the IOP was 10 [8, 13] mmHg (*p* = 0.002) on 0 [0.0, 3.0] topical medications. The median BCVA was 0.2 [1.0, −0.1] logMAR and 0.2 [1.0, −0.2] logMAR before and after surgery respectively. Out of these 80 eyes, 68% (*n* = 54) were phakic and 32% (*n* = 26) were pseudophakic. Ten patients (14%) underwent cataract surgery after trabeculectomy, but *none within one year*.

**Fig. 2:**
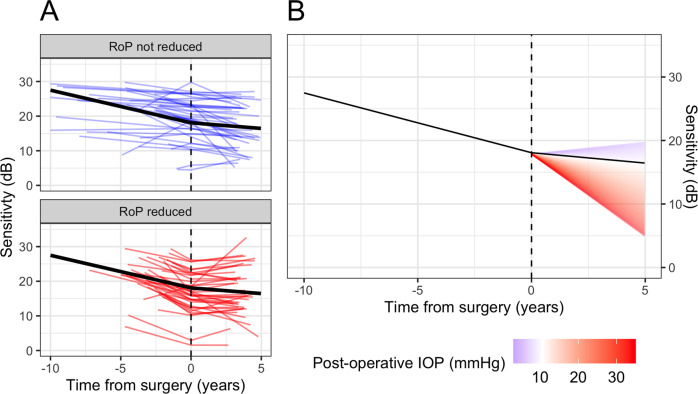
Average predicted RoP before and after surgery (black solid line). **A** The time of surgery is marked by a vertical dashed line. The smaller coloured lines indicate the fits on individual eyes, estimated from the random effects. Eyes with a significant reduction in RoP are in red. **B** Average predicted RoP before and after surgery is represented by a black solid line (same as **A**). The coloured gradient represents how the estimated post-operative RoP changes based on the IOP at one year from surgery. RoP Rate of progression, IOP Intraocular pressure.

The mean RoP before surgery was −0.94 [−1.20, −0.69] dB/year (Mean [95% Credible Intervals]) and it was slowed down by 0.62 [0.26, 0.97] dB/year (p_d_ < 0.001, Fig. 2A) to −0.33 [−0.57, −0.08] dB/year after surgery. The difference in RoP before and after surgery was positive for 60/80 (75%) of the eyes, but significant only in 48/80 eyes (60%). There was a statistically significant effect of the post-operative IOP at one year from surgery on the amount of RoP reduction (−0.09 [−0.14, −0.04] dB/year of RoP change per mmHg, p_d_ < 0.001, Fig. 2B). The predicted RoP change with 10 mmHg post-operative IOP was 0.71 [0.40, 1.02] dB/year. However, there was no significant effect when IOP was modelled as difference or percentage change from listing IOP (p_d_ = 0.682 and p_d_ = 0.627 respectively).

## Discussion

Trabeculectomy is known as the standard filtering surgery for uncontrolled glaucoma. Its beneficial effect of IOP reduction has been demonstrated in several studies. Nevertheless, despite surgery, many glaucoma patients still show progression of VF defects. The aim of this study was to move away from typically assessed outcome parameters after surgery (i.e. IOP) and to focus on visual function outcomes.

Our results provide evidence that surgically induced IOP-lowering results in a markedly decreased RoP with significant IOP-reduction and minimization of topical IOP-lowering agents without aiming for a specific postoperative IOP level. In our cohort, 75% of the patients had a post-operative IOP ≤ 13 mmHg. Still, the small variability among patients was enough for us to detect a significant effect of IOP control at one year on the RoP. The average RoP reduction was statistically significant and clinically relevant. In simple terms, reducing the ROP by 0.62 dB/year means a saving of 6.2 dB of VF loss for the patient’s next decade of life. Of course, there are several caveats with this extrapolation, but a reduction of this magnitude could affect a person’s vision-related quality of life, *saving sight years* [20].

Trend analyses, which analyse changes in VFs by using serial measurements to determine the RoP, often involve linear regression of summary statistics like mean deviation (MD). However, rates of VF deterioration are not necessarily constant over time due to patients’ compliance to treatment or change in treatment intensity. Nevertheless, most rates of VF loss are typically well described by a linear decay. Many factors contribute to deciding whether a specific rate of progression is clinically important: the current stage (severity) of disease, the life expectancy of the patient and the severity/stage at which the person-specific vision-related quality of life would be affected. It is important to note that the effect of VF loss on quality of life also greatly depends on the specific pattern of loss and asymmetry of VF damage [21]. While our methodology could be extended to explore the topography of VF loss, this was beyond the scope of the current analysis. In our cohort, at surgery, the median MD was −10.84 dB. This means that, on average, a change in RoP from −0.94 dB/year to −0.33 dB/year will extend their time to visual impairment, defined as a MD < 22 dB [22], from 12 to 34 years, well beyond the expected remaining life span at the age of surgery (median 67 years).

It is important to note that some patients might have been listed for surgery because the IOP was not deemed ‘at target’ for the level of VF without a documented progression of damage. This might have biased the pre-operative slopes. However, this might have diluted the overall measured benefit of surgery, which might be even greater.

The Early Manifest Glaucoma Trial reported a median rate of MD loss in untreated eyes of −0.4 dB/year in 118 glaucoma patients [23]. However, there appears to be a large variability in rates of change in VFs in glaucoma patients. Median rates of loss in treated clinical practice differ widely and show a range from −0.05 dB/year to −0.62 dB/year [24, 25]. Different studies reported a change in visual field index (VFI) in glaucoma patients between −1.1 and 1.5%/year [26, 27]. Our data show a mean loss of sensitivity of −0.94 dB/year before surgery, compatible with a moderate to fast progression. Because we modelled VF sensitivity and not age corrected metrics, our estimates of progression include the effect of normal aging (reported to be −0.64 dB/decade, 95% CI, −0.74 to −0.53 dB/decade) [28]. It should be noted that such a small effect would be negligible over the relatively short time span considered in our analysis and would have very little bearing on the comparison of RoP before and after surgery.

The beneficial effect of surgically induced IOP-lowering by means of trabeculectomy has been demonstrated in different studies, with success defined by the amount of IOP reduction achieved [22]. However, around 13 – 83% of the cases still show some progression after surgery [6, 7]. The progression rate of eyes treated medically or surgically seems to be the same, if there is a similar IOP outcome [3]. Studies report greater IOP reduction associated with better preservation of visual fields after surgery [29, 30]. A specific level of desirable IOP is not yet established. Mao et al. found that, in patients with early glaucomatous damage and POAG undergoing medical treatment or laser trabeculoplasty, all eyes with IOP < 21 mmHg during their follow-up demonstrated progressive glaucomatous changes. On the contrary, eyes with an IOP of <17 mmHg remained stable [31]. The AGIS study with more advanced disease, on the other hand, showed that most eyes with an IOP < 18 mmHg over the first six follow-up years had stable field defect scores but still about 14% of eyes had considerable field loss at 5–7 years, despite having an IOP < 18 mmHg at all study visits [2]. In our cohort, one year after surgery, the median IOP was 10 mmHg (*p* < 0.01). Nevertheless, comparing different studies is difficult since progression is defined using various criteria. Many studies have compared criteria used in clinical trials (typically event based definitions of progression) and demonstrated that the proportion of progressing series varies greatly.

Palmberg suggested a plausible explanation for these contradictory findings regarding the role of the postoperative IOP level and assumed that treatment was more likely to be aggressive in patients who seem to be at greater risk of progression [32].

Besides IOP level, other risk factors for faster progression have been identified and could contribute to the findings. Studies show that increasing age, worse baseline VF damage, higher baseline IOP and the presence of conditions such as exfoliation can all have an impact [3, 31]. Other risk factors for progression include bilateral disease, worse mean deviation and frequent disc haemorrhages during follow-up [33].

More recent studies also found evidence that VFs might improve after surgery [9, 10]. Electrophysiologic studies indicated improvement after IOP reduction, suggesting reversibility of retinal ganglion cell (RGC) function [34, 35], but our study was not designed to investigate this aspect as such an improvement is often reported to be transient in nature [22].

Our cohort of patients was a random selection from our clinics with heterogeneous characteristics. All were identified as candidates for trabeculectomy and on average achieved excellent mean IOP control post-op. Our analysis confirms the implication of such a reduction on the preservation of VF.

Our study is limited by its retrospective approach; we have no control arm. The greatest caveat must be made about the possibility of regression to the mean because most of the patients analysed are likely to have been listed for surgery based on their progression of VF damage. If this was caused by a few poor test performances in the pre-operative VF series, regression to the mean might explain an apparent post-operative improvement. This effect is reduced, but not eliminated, by our two-stage regression model, which forces the pre and post-operative regression lines to connect, dampening the effect of outliers near the date of surgery. This can be appreciated in the example reported in Fig. 1A, especially in some locations in the superior-nasal quadrant. For example, the third location in the second row in Fig. 1A shows one observation clearly dropping in sensitivity before surgery. However, this drop is not sustained after surgery. This would have greatly affected the estimate of the pre-operative RoP. This is mitigated by our model forcing the two lines to meet at the day of surgery. Another source of bias can arise from the floor effect in VF tests, which would necessarily be more pronounced in the post-operative phase for progressing patients as sensitivity values would be closer to 0 dB. This was however specifically addressed by our methodology (Fig. 1B), and this is novel. The 72 (36.2%) eyes excluded because of insufficient pre-operative VF data were listed for surgery early after their glaucoma diagnosis or referred from another hospital for surgery without visual field tests available for analysis. Another confounder might be the effect of progressing cataract after surgery. However, this would work against our main results, showing a global VF decline independent of glaucomatous damage. One way of accounting for this would be to use pattern deviation values to monitor progression. However, this would have prevented us from correctly modelling the floor effect and would not be appropriate for many of the patients in this cohort owing to their advanced VF damage. Finally, a longer pre and postoperative follow-up with more reliable VFs would be desirable.

In conclusion, this data provides information to clinicians, surgeons and patients about expected visual function outcomes without focusing on surrogate outcomes such as IOP.

### Summary

#### What was known before

- Lowering the IOP by means of trabeculectomy has a great impact on the preservation of the visual field.
- The effectiveness on the rate of progression has only been partially investigated.

#### What this study adds

- This study adds data on expected visual function outcomes to clinicians, surgeons and patients without focusing on surrogate outcomes, i.e. intraocular pressure.
- It provides evidence that surgically IOP-lowering results in a markedly decreased rate of progression of the visual field without aiming for a specific postoperative IOP level.

## Supplementary information

Supplementary material

## Data Availability

All data generated or analysed during this study are included in this published article (and its supplementary information files).
