# Supplementary material for: Effect of trabeculectomy on the rate of progression of visual field damage
Source: Eye (Lond). 2022 Dec 7;37(10):2145–50. doi: 10.1038/s41433-022-02312-y (PMC10333233; doi:10.1038/s41433-022-02312-y)
Supplement: Supplementary file 1 — Supplementary material [file 41433_2022_2312_MOESM1_ESM.docx]

**Supplementary to: Effect of trabeculectomy on the rate of progression of visual field damage**

Susanna Friederike Koenig^1,2^, Giovanni Montesano^3,4^, Clarissa Ern Hui Fang^1^, David Paul Crabb^3^, Hari Jayaram^1,4^, Jonathan Clarke^1,4^

^1^Moorfields Eye Hospital NHS Foundation Trust, 162 City Road, EC1V 2PD, London, United Kingdom

^2^Universitaetsaugenklinik Ulm, Prittwitzstrasse 43, D – 89075 Ulm, Deutschland, Germany

^3^Optometry and Visual Science, City University of London, London, UK

^4^NIHR Biomedical Research Centre of Ophthalmology, Moorfields Eye Hospital and UCL Institute of Ophthalmology, London, UK

# **Bayesian modelling**

## *Linear broken-stick model*

The broken-stick model has sensitivity over time as its response variable and two hierarchical levels: eye and location. These two levels are modelled as nested random effects. The rate of progression (RoP) and the effect of surgery are modelled using fixed effects. The RoP corresponds to the coefficient assigned to the effect of time on VF sensitivity. Time was expressed in years from surgery, assuming negative values prior to surgery and positive values after surgery, so that the break-point was the same for all subjects (day of surgery, i.e. 0). The effect of surgery on the rate of progression was modelled by an additional fixed effect. This was a quantity identical to time, but all negative values (i.e. time prior to surgery) were set to 0. This means that such an effect would be non-zero, and additive, only after surgery. The coefficient for this effect represents the difference in RoP before and after surgery. The fixed effect portion of the model is reported below:

$${Sensitivity}_{dB}=\beta_{0}+\beta_{1}*Time +\beta_{2}*{Time}_{Post-surgery}$$

In this equation, $\beta_{1}$ represents the RoP (in dB/year) of sensitivity over time, while $\beta_{2}$ represents the change in RoP after surgery. Therefore, the RoP after surgery will be given by the sum of $\beta_{1}$ and $\beta_{2}$. This entails that, if $\beta_{2}$ is equal to zero, the RoP does not change after surgery. Hence, the value of $\beta_{2}$ is our *outcome of interest* and is the one tested for significance in the main analysis. VF sensitivity was assumed to have a normal distribution of the residuals, censored at 0 dB, to account for the floor effect in the measurement.

Fitting of the Bayesian model was achieved using JAGS (Just Another Gibbs Sampler^1^) to run Markov Chain Monte Carlo (MCMC) simulations, within the R environment (R Foundation for Statistical Computing). Two parallel MCMCs were run for at least 50,000 iterations (thinning = 10 iterations) after 1,000 adaptation steps and 5,000 burn-in iterations. Only chains for the fixed effect parameters and the eye level random effects were monitored. The MCMCs were stopped if the Gelman-Rubin diagnostic was < 1.2 for all the monitored parameters, indicating convergence^2^. Prior distributions on the fixed effects were non-informative Normal distributions with a precision of 0.01 (Variance = 100) and the mean determined by the results of a frequentist fit on the same data (using the package *lme4*^3^, reported later). These were also the starting values for the MCMCs, to optimize the initial sampling of the posterior distribution. The joint prior distribution for the random intercept and slope at each hierarchical level was a bivariate Normal distribution with zero mean and a 2 x 2 precision matrix. The prior for the precision matrix was a Wishart distribution with a 2 x 2 inverse variance matrix with 0 off diagonal values and 2 degrees of freedom.

## *Effect of intraocular pressure*

A modification of the model used for the primary outcome analysis was implemented to analyze the effect of the intraocular pressure (IOP) at one year after surgery. Simply, an additional parameter was added to the equation, consisting of the effect of time post-surgery multiplied by the value of IOP. The random effect structure and the fitting procedure was the same as for the main model. The fixed effect portion of this second model is reported below:

$${Sensitivity}_{dB}=\beta_{0}+\beta_{1}*Time +\beta_{2}*{Time}_{Post-surgery}+ \beta_{3}*{IOP*Time}_{Post-surgery}$$

In this equation, $\beta_{3}$ represents the effect of the IOP at one year from surgery on the post-operative change in RoP. Therefore, the change in RoP after surgery needs to be considered in conjunction with the IOP. For example, the change in RoP after surgery for an eye in which a post-operative IOP of 10 mmHg was achieved would be calculated as $\beta_{2}+ \beta_{3}*10$. As before, a value of $\beta_{3}$ equal to 0 would indicate no effect of post-operative IOP on the post-operative change in RoP, and this was our null hypothesis for testing this parameter.

## *P-direction and hypothesis testing*

The P-direction was calculated as in Makowski et al.^5^. Briefly, the median of the posterior distribution is determined from the MCMC draws. Taking for example the difference in the RoP between the two arms of the trial (primary outcome, see **Figure S1**), the assumed null hypothesis is that of no difference (i.e., difference = 0 dB/year). According to whether the median is above or below 0, the proportion of MCMC draws above or below 0 is the P-direction, respectively. Therefore, such a quantity can have values between 0.5 (distribution centered on 0) and 1. The p_d_ statistic can then be calculated as $p_{d} =2*(1 - P-direction)$^5^ and has a similar interpretation to a frequentist two-tailed p-value.


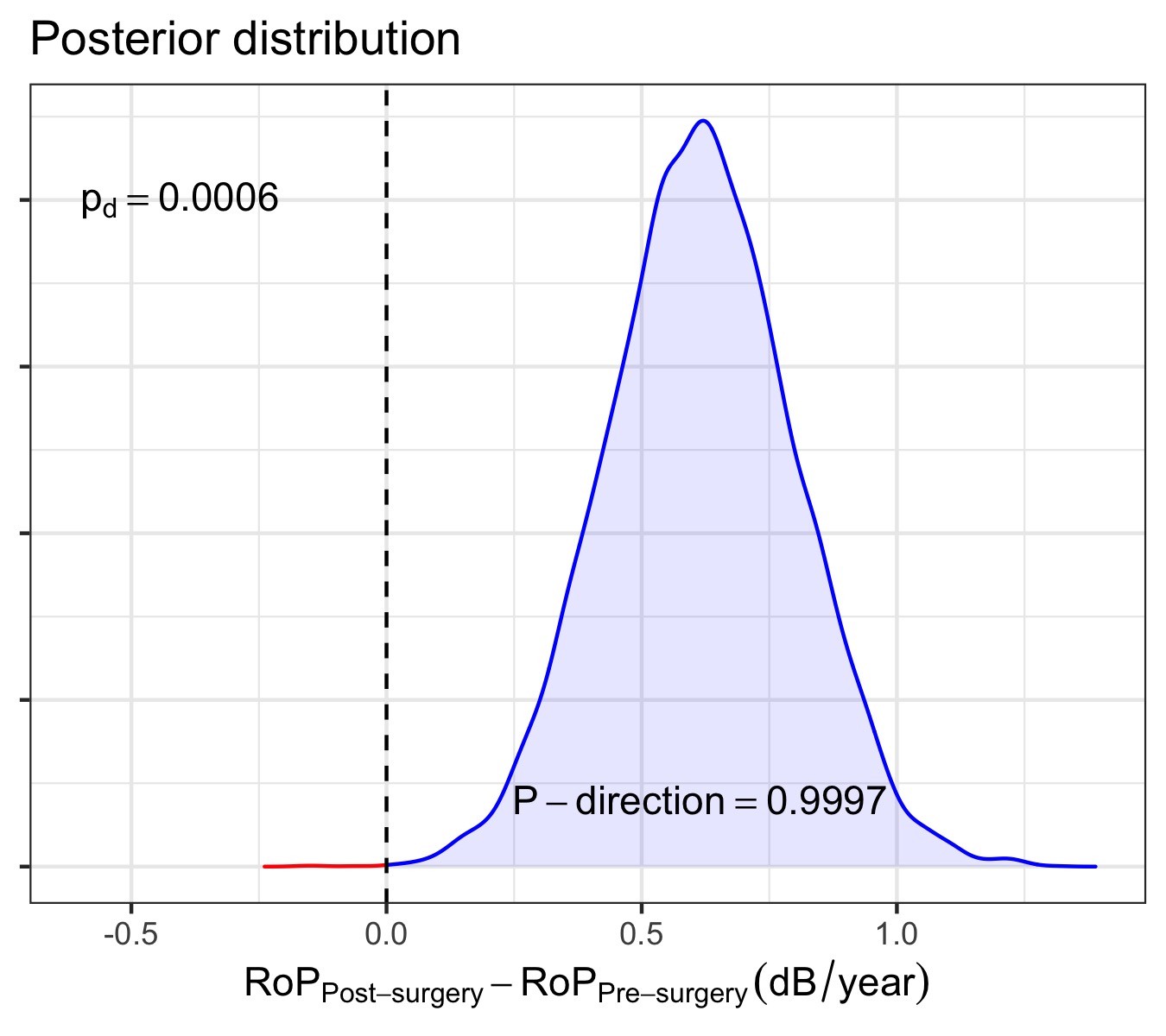


**Figure S1.** Density estimation of the posterior distribution for the MCMC draws for the difference in RoP pre and post-surgery. The vertical dashed line represents the assumed null hypothesis (i.e. no difference). The P-direction is the proportion of observations above 0 dB/year (area shaded in light blue). RoP = Rate of Progression.

# *Frequentist modelling*

A frequentist version of the model used for the main analysis was fitted using the *lme4* package for R^3^, without accounting for censoring. The mean RoP before surgery was -0.84 [-1.08, -0.61] dB/year (Mean [95% Confidence Intervals]) and it was slowed down by 0.57 [0.25, 0.90] dB/year (p < 0.001).

The effect of post-operative IOP on the change in RoP estimated with the frequentist approach was (-0.09 [-0.13, -0.04] dB/year of RoP change per mmHg, p < 0.001).

# **References**

1. Plummer M. JAGS: A program for analysis of Bayesian graphical models using Gibbs sampling. 2003.

2. Andrew G, Donald BR. Inference from Iterative Simulation Using Multiple Sequences. Statistical Science 1992;7(4):457-72.

3. Bates D, Mächler M, Bolker B, Walker S. Fitting Linear Mixed-Effects Models Using lme4. Journal of Statistical Software; Vol 1, Issue 1 (2015) 2015.

4. Garway-Heath DF, Poinoosawmy D, Fitzke FW, Hitchings RA. Mapping the visual field to the optic disc in normal tension glaucoma eyes. Ophthalmology 2000;107(10):1809-15.

5. Makowski D, Ben-Shachar MS, Chen SHA, Ludecke D. Indices of Effect Existence and Significance in the Bayesian Framework. Front Psychol 2019;10:2767.
